# 71461 Intimate Partner Violence and HIV Testing among Women in Rural Southwestern Uganda

**DOI:** 10.1017/cts.2021.736

**Published:** 2021-03-30

**Authors:** Cassandra Schember, Jessica Perkins, Viola Nyakato, Bernard Kakuhikire, Allen Kiconco, Betty Namara, Lauren Brown, Carolyn Audet, April Pettit, David Bangsberg, Alexander Tsai

**Affiliations:** 1 Vanderbilt University; Viola Nyakato, Mbarara University of Science and Technology; 2 Mbarara University of Science and Technology; 3 University of Witwatersrand; 4 MRC/UVRI & LSHTM Uganda Research Unit; 5 Nashville CARES; 6 Oregon Health & Science University-Portland State University School of Public Health; 7 Harvard Medical School

## Abstract

ABSTRACT IMPACT: This research shows that physical intimate partner violence was associated with never testing for HIV while verbal intimate partner violence was associated with increased testing for HIV suggesting that HIV testing interventions should consider intimate partner violence prevention. OBJECTIVES/GOALS: HIV incidence is higher among women who experience intimate partner violence (IPV). However, few studies have assessed the association between HIV testing (regardless of the result) and the experience of IPV. Our objective was to assess the relationship between IPV and HIV testing among women from rural southwestern Uganda. METHODS/STUDY POPULATION: We conducted a whole-population, cross-sectional study including women ?18 years of age who were permanent residents in 8 villages of Rwampara District, southwestern Uganda from 2011-2012 who reported having a primary partner in the past 12 months. We surveyed participants to assess their exposure to 12 different forms of verbal, physical, and/or sexual IPV, and whether they had ever been tested for HIV. We used three separate modified Poisson regression models, clustering by village, to estimate the association between each type of IPV and ever testing for HIV, adjusting for categorical age, completion of more than primary education, and any food insecurity measured by the nine-item Household Food Insecurity Access Scale. RESULTS/ANTICIPATED RESULTS: Among 496 women with a primary partner (>95% response rate), 64 (13%) had never tested for HIV, 297 (60%) reported verbal IPV, 81 (16%) reported physical IPV, and 131 (26%) reported sexual IPV. Further, among these women, 208 (42%) were aged <30 years, 378 (76%) had a primary or no education, and 390 (79%) experienced food insecurity. Never having been tested for HIV was positively associated with physical IPV (adjusted risk ratio (ARR): 1.61, 95% confidence interval (CI): 1.02-2.56) and negatively associated with verbal IPV (ARR: 0.67, 95% CI: 0.44-0.99), but not sexual IPV (ARR: 1.05, 95% CI: 0.51-2.12). DISCUSSION/SIGNIFICANCE OF FINDINGS: Among this population of adult women with partners in Uganda, physical IPV was associated with never testing for HIV while verbal IPV was associated with increased testing for HIV. Evidence suggests that HIV testing interventions should consider IPV prevention, and future studies should focus on why certain IPV types impact HIV testing rates.

